# Vitamin D deficiency among pregnant women in India

**DOI:** 10.6026/9732063002002029

**Published:** 2024-12-31

**Authors:** Adawadkar Nitish, Patidar K Anand, Likhar Swarnkanta, Pandey Dhruvendra

**Affiliations:** 1 Department of Community Medicine, Government Medical College, Ratlam, Madhya Pradesh, India

**Keywords:** Vitamin D, cholecalciferol, pregnancy, fetal growth, maternal-fetal relations, antenatal care

## Abstract

Vitamin D is a fat-soluble vitamin involved in the regulation of calcium homeostasis and thus is essential for a healthy skeletal system. The blood samples from pregnant females coming at the facility were tested for Vitamin D levels as a part of routine Ante natal care (ANC). We report that 130 (61.9%) pregnant women had insufficient Vitamin D levels (12-20ng/mL) and 56 (26.6%) pregnant Women were having Vitamin D Levels (20ng/mL). Moreover, the mean Vitamin D (ng/mL) level in pregnant women was 14.3 ±5.2 ng/ml. Thus, there was a deficiency of vitamin D in most of the ANC mothers registered in UHTC. Hence, adequate supplementation of Vitamin D is needed during pregnancy. Lack of awareness is the major risk factors associated with Vitamin D insufficiency.

## Background:

Vitamin D is a fat-soluble vitamin involved in the regulation of calcium homeostasis and essential for a healthy skeletal system [1-2]. It also acts as a pro hormone. It exists in two forms Vitamin D2 (ergocalciferol) and vitamin D3 (cholecalciferol) [1]. It is essential for a better immune system and brain development of fetus [3]. Vitamin D deficiency during pregnancy is the most important risk factor for childhood rickets and can also lead to poor fetal growth and neonatal development [4]. Furthermore, vitamin D deficiency in pregnant women can lead to gestational diabetes and preeclampsia [3, 4-5]. There is a rising incidence of vitamin D deficiency among pregnant women worldwide. People obtain vitamin D (cholecalciferol) through exposure to sunlight, diet and supplements [3]. Only few foods contain or are rich in vitamin D (liver, fatty fish, eggs, milk and dairy products, soy milk, butter, margarine, *etc.* hence the cutaneous synthesis of vitamin D induced by ultraviolet B radiation (UVB) is the most important source for the vitamin D [6]. Level less than 12 ng/ml Deficient, 12-20 ng/ml insufficient and >20 ng/ml is considered optimal [7]. Several studies have shown a high preponderance (ranging from 3% to 86 %) of Vitamin D deficiency among pregnant women [8-9]. In tropical country like India where most of the population receives abundant sunlight throughout the year, there is widespread prevalence of Vitamin D deficiency. As per a study conducted in India on pregnant women 84% were having Vitamin D level <22.5 ng/ml [9]. The recommended daily intake of vitamin D ranges from 400 to 600 IU (by the IOM), 400 IU (by the National Institute for Health and Clinical Excellence, United Kingdom) and to 1500-2000 IU (by the Endocrine Society) and 2000 IU (by the Canadian Society) [10]. Results of the recently conducted randomized controlled trial on vitamin D supplementation in pregnancy suggest a safe dose of 2000-4000 IU/day [11]. The daily upper safe limit for vitamin D has been set at 4000 IU by IOM and 10,000 IU by the Endocrine Society [12]. Therefore, it is of interest to show the Vitamin D status among pregnant women and its association with maternal age, parity, Vitamin D supplementation.

## Methodology:

This cross - sectional study was carried out in 210 pregnant women registered in Urban Health & Training Centre at Ratlam, India. The study was conducted between 1^st^ January 2023 -30th April 2023 after the clearance from Institutional Ethical Committee. As per study done by Raut *et al.* in 2022 at Khargar, the prevalence of vitamin D deficiency in pregnant women was 85%. After applying the formulae 4pq/l^2^., taking 5% as allowable error and 95% confidence level, the sample size comes out to be 210. Based on that the sample size came out were 210. This sample size was distributed in 60 days of sample collection. Per day first 4-5 (210/50) pregnant women coming to UHTC for ANC check-up were taken in this study as per consecutive sampling method. Pregnant women were explained about the purpose of the study and informed consent was obtained. Pregnant women with known comorbidities such as essential hypertension, diabetes, thyroid disease, renal disease, or any other medical illness, were excluded from the study. Blood samples were collected with prior consent and subsequently analyzed for vitamin D levels, in addition to standard tests including HIV screening and haemoglobin assessment, in accordance with government protocols and under aseptic conditions. A comprehensive history encompassing personal details, medical history and medication usage (specifically iron and folic acid as well as vitamin D intake) was obtained through the use of open-ended questionnaires. Venous blood samples from the participants were evaluated for vitamin D and serum calcium levels using chemiluminescence immunoassay techniques at the biochemistry laboratory of the UHTC. Haemoglobin was estimated using the card test. Blood pressure was measured using sphygmomanometer. Pre testing of tentative validated questionnaire (Hindi/local language) was done in 20 pregnant women registered in Urban Health & Training Centre Ratlam before starting of the project. After data collection, the data was entered into Microsoft Office Excel and analyzed using EpiInfo 7, free software.

## Results:

## Antenatal profile of the study population:

Out of total 210 pregnant women 72 (34.2%) were in 1st trimester, 91(43.3%) women were in 2nd trimester and 47 (22.3%) women were in 3rd trimester. 138 (65.7%) women were first time pregnant (primigravida) and 72 (34.2%) women were multigravida. The Mean Age (years) of the pregnant women was 28 ± 5.1 years. Among the pregnant women studied, 130 (61.9%) exhibited insufficient Vitamin D levels, falling within the range of 12-20 ng/ml. Additionally 56 (26.6%) had Vitamin D levels below 12 ng/mL while 24 (11.4%) were classified as having sufficient levels, exceeding 20 ng/ml. The average Vitamin D level recorded was 14.3 ± 5.2 ng/ml.

## Socio demographic characteristics of the study population:

As per this study 16 (7.6%) women were postgraduate's, 54 (25.7%) pregnant women were graduate's and 29 (13.8%) were illiterate. 111 (52.8%) had completed their education up to intermediate/diploma level. 142 (67.6%) pregnant women belonged to lower middle-class family and 68 (32.3%) pregnant women belonged upper middle-class family. Out of 210 Pregnant women 147 (70%) were non vegetarian and 63 (30%) were vegetarian.

## Clinical profile of the study population:

Mean vitamin D level in the deficient group was 8.1 ± 1.06 ng/mL and this level was significantly lower when compared to the insufficient (14.4±2.3 ng/mL) and sufficient group (27.2 ± 8.25 ng/mL; p<0.02). The mean B.M.I (kg/m^2^) of 210 pregnant women came out to be 29.8 ± 4.2. The mean SBP (mmHg) in Vitamin D Deficient pregnant women was 107 ±12.1, 109 ±10.5 (mmHg) in Vit D insufficient mothers and 112 ± 9.8 (mmHg) in Vit D sufficient pregnant women and no statistical significant difference noted (P value >0.05).The Mean DBP (mmHg) was 65 ±7.6 in Vit D Deficient pregnant women , 71 ± 10.2 (mmHg) in pregnant women with Vit D insufficiency and 76 ± 8.8 (mmHg) in pregnant women having sufficient Vitamin D on applying statistical test no statistical significant difference was noted (P value >0.05). Among the Vit. D sufficient, Insufficient and Deficient groups no statistically significant difference was noted with reference to Haemoglobin and Random Blood Sugar levels (P value >0.05). Among the first trimester females 20 (27.8%) were vitamin D deficient, 44 (61.1%) were Vitamin D insufficient and 8 (11.1%) had sufficient vitamin D levels. In the second trimester females, 7 (7.7%) were vitamin D deficient and 10 (11%) had sufficient vitamin D levels. Among the total 47 third trimester females, the percentage of vitamin D deficiency was 61.7% (29 females) which is quite high and only 6 (12.8%) had sufficient vitamin D levels as shown in Figure 1 (see PDF).

## Discussion:

Vitamin D deficiency is a widespread yet often neglected nutritional issue globally. In India, despite ample sunlight, it remains a growing public health concern. Various factors influence the body's Vitamin D production, including limited sun exposure, air pollution, skin conditions, activity levels and clothing and sun protection habits [13]. Vitamin D deficiency leads to adverse health problems in pregnant women and their newborns. Increased preponderance of Vitamin D deficiency in pregnant women has been reported from different countries, ranging from 45% to 100% [14]. A prevalence of 26.6% of Vitamin D deficiency and 61.9% of Vitamin D insufficiency was observed among pregnant women in this study which is similar to finding in tropical countries like India where deficiency percentage ranges between 42%-93%. [7, 8, 9, 10, 11, 12, 13, 14-15]. It was reported by Nageshu et al that 53.8% of Third trimester women had insufficient Vitamin D [16]. Our result show that 61.7% of 3rd Trimester women were deficient in Vitamin D which is similar to a study done by Shehla *et al.* (2020) where Vitamin D insufficiency was found in 67.4% of the pregnant women [17]. In this study mean vitamin D level in Vitamin D deficient women was 8.1 ± 1.06 (ng/mL) and in Vitamin D insufficient pregnant women it was 14.4 ± 2.3 (ng/mL). The mean Vitamin D levels in Vitamin D sufficient pregnant women were 27.2 ± 8.5 (ng/mL). A study done by Goswami *et al.* (2000) reported the mean concentration of serum 25(OH) D in pregnant women to be 8.6 ± 4.28 (ng/ml) [18]. Mean B.M.I (kg/m^2^) of 210 pregnant women was 25.8 ± 4.2 years. As per the findings of Ravinder *et al.* (2022) mean B.M.I in Vitamin D insufficient pregnant women was 26.36 ± 2.7 and 25.84 ± 3.6 in Vitamin D adequate pregnant women [19].

The socio-economic status of pregnant women showed a significant correlation with their vitamin D levels. According to the research conducted by Ravinder *et al.* (2022), there was a strong statistical association between the socioeconomic status of these women and their vitamin D levels [19]. Similar findings were seen in the study conducted by Sharma *et al.* (2016) [20]. The deficiency and insufficiency of Vitamin D were observed to be more prevalent in nulliparous women than in multiparous women. No statistically significant correlation was found between Vitamin D levels and random blood sugar. However, prior research has indicated that the relationship between Vitamin D levels and gestational diabetes mellitus is influenced by the ethnicity of the study population [21]. There was no significant association seen between Vitamin D levels and Haemoglobin level in pregnant women. As per previous study done in China shown the levels of plasma Vitamin D in 1^st^, 2^nd^ and 3^rd^ trimester were positively associated with Haemoglobin levels [22]. A cross-sectional study in Sudan that found no correlation between serum Vitamin D and Hb level [23]. The possible reason for this inconsistency could be different geographical conditions, socio-economic status *etc.* The consumption of iron and folic acid supplements was higher than that of vitamin D supplements, primarily because Iron folic acid tablets are provided free of charge at the UHTC and are accessible in all public hospitals. The lower intake of vitamin D supplements can be attributed to a lack of awareness regarding their importance, limited availability of vitamin D supplements at the UHTC and the fact that these supplements are predominantly prescribed to patients with orthopedic issues, such as arthritis and back pain, as well as to elderly patients. The number of pregnant women who received Vitamin D testing in the last 12 months was lower than those who did not undergo the test. This difference may be due to the significant cost of testing, which varies between approximately 800 to 1500 INR, as Vitamin D is not included in the standard Antenatal Care (ANC) profile typically offered in public hospitals. Nevertheless, certain private practitioners do advise Vitamin D testing for their pregnant patients.

## Conclusion:

Vitamin D deficiency is notably common among pregnant women enrolled at the Urban Health Training Centre in Ratlam, India. Low socioeconomic status, a lack of nutritional awareness and insufficient vitamin D supplementation during pregnancy are contributing factors.

## Limitations:

The evaluation of dietary consumption and vitamin D supplementation as reported by the pregnant participants may be influenced by recall bias. The other variables such as skin complexion, sun exposure (duration), seasonal variation *etc.*, we're not taken in to consideration.

## Recommendation:

It is essential to incorporate Routine Vitamin D screening into maternal and child health programs. Insufficient Vitamin D can be addressed by fortifying foods or using supplements, available as tablets, capsules, or syrup. In shaping future policy, research must assess several key factors. These factors encompass the extent to which serum Vitamin D levels enhance maternal and offspring health outcomes across diverse dietary habits and skin pigmentation, determine the optimal timing for initiating therapy, as well as the most effective and safe dosage.

## Source of funding:

No funding source
